# Consolidated bioprocessing performance of *Thermoanaerobacterium thermosaccharolyticum* M18 on fungal pretreated cornstalk for enhanced hydrogen production

**DOI:** 10.1186/s13068-014-0178-7

**Published:** 2014-12-24

**Authors:** Lei Zhao, Guang-Li Cao, Ai-Jie Wang, Hong-Yu Ren, Kun Zhang, Nan-Qi Ren

**Affiliations:** State Key Laboratory of Urban Water Resource and Environment, Harbin Institute of Technology, Harbin, 150090 China; School of Life Science and Technology, Harbin Institute of Technology, Harbin, 150090 China; College of Power and Energy Engineering, Harbin Engineering University, Harbin, 150001 China

**Keywords:** Consolidated bioprocessing, *Thermoanaerobacterium thermosaccharolyticum* M18, Pretreatment, Hydrogen production, Cornstalk

## Abstract

**Background:**

Biological hydrogen production from lignocellulosic biomass shows great potential as a promising alternative to conventional hydrogen production methods, such as electrolysis of water and coal gasification. Currently, most researches on biohydrogen production from lignocellulose concentrate on consolidated bioprocessing, which has the advantages of simpler operation and lower cost over processes featuring dedicated cellulase production. However, the recalcitrance of the lignin structure induces a low cellulase activity, making the carbohydrates in the hetero-matrix more unapproachable. Pretreatment of lignocellulosic biomass is consequently an extremely important step in the commercialization of biohydrogen, and for massive realization of lignocellulosic biomass as alternative fuel feedstock. Thus, development of a pretreatment method which is cost efficient, environmentally benign, and highly efficient for enhanced consolidated bioprocessing of lignocellulosic biomass to hydrogen is essential.

**Results:**

In this research, fungal pretreatment was adopted for enhanced hydrogen production by consolidated bioprocessing performance. To confirm the fungal pretreatment efficiency, two typical thermochemical pretreatments were also compared side by side. Results showed that the fungal pretreatment was superior to the other pretreatments in terms of high lignin reduction of up to 35.3% with least holocellulose loss (the value was only 9.5%). Microscopic structure observation combined with Fourier transform infrared spectroscopy (FTIR) analysis further demonstrated that the lignin and crystallinity of lignocellulose were decreased with better holocellulose reservation. Upon fungal pretreatment, the hydrogen yield and hydrogen production rate were 6.8 mmol H_2_ g^-1^ pretreated substrate and 0.89 mmol L^-1^ h^-1^, respectively, which were 2.9 and 4 times higher than the values obtained for the untreated sample.

**Conclusions:**

Results revealed that although all pretreatments could contribute to the enhancement of hydrogen production from cornstalk, fungal pretreatment proved to be the optimal method. It is apparent that besides high hydrogen production efficiency, fungal pretreatment also offered several advantages over other pretreatments such as being environmentally benign and energy efficient. This pretreatment method thus has great potential for application in consolidated bioprocessing performance of hydrogen production.

## Background

Lignocellulosic biomass is the most abundantly available raw material on the earth [1]. In China alone, for example, the annual yield of cornstalk is up to 220 million tons. Unfortunately, most of the biomass residuals are disposed of by direct combustion owing to lack of effective utilization. In order to strive for proper use of this large amount of resource, production of biofuels from lignocellulosic biomass should be given increasing amounts of attention [2]. Hydrogen gas (H_2_) is currently one of the most promising alternatives to conversional fossil fuels because of its desirable characteristics such as high calorific value and its renewability [3,4]. Hence, biological H_2_ production from lignocellulosic biomass such as cornstalk can not only help utilize this resource rationally, but also reduce the earth’s dependence on fossil fuels. Biological conversion of lignocellulosic biomass into H_2_ commonly involves i) pretreatment of lignocelluloses, ii) hydrolysis of polysaccharide constituents into reducing sugars with a cellulolytic enzyme cocktail, and iii) fermentation of sugars with H_2_-producing microorganisms. Traditionally, these processes were carried out at different reactors, or by different microorganisms separately [5]. In recent years, an alternative approach known as consolidated bioprocessing (CBP), which could combine enzyme production, saccharification, and H_2_ fermentation in a single step was developed. This integrated process configuration is more favorable for cellulosic H_2_ production due to its simple operating process, low capital and substrate cost, short processing time, high H_2_ yields, and low contamination risk [6-9]. Consequently, CBP is regarded as the optimal industrial configuration to produce H_2_.

The thermophilic bacterium *Thermoanaerobacterium thermosaccharolyticum* M18 shows great ability in producing H_2_ directly from various defined polycarbohydrates such as xylan, filter paper, Avicel, and a variety of natural lignocellulosic materials such as corn cob, corn stover, and wheat straw [10], and thus it is regarded as a promising candidate CBP microbe for biohydrogen production from lignocellulosic biomass. However, an appreciable decrease in H_2_ yield was observed with natural cellulosic substrates compared with pure cellulose substrate [10]. This finding indicated that the strain M18 can utilize natural substrates, but not as efficiently as pure cellulose. According to the previous studies, the crystallinity and heterogeneity of the lignocellulose structure usually induced low cellulolytic activity and slow specific growth rates of the microorganisms involved [11,12]. As a result, an appropriate pretreatment to break the lignin barrier and disrupt the crystalline structure of lignocellulosic biomass is crucial to improve the accessibility of holocellulose (cellulose and hemicellulose) in the process of CBP to the microorganism and thus enhance cellulosic biohydrogen production.

Thermochemical pretreatment is the most widely used method for pretreatment of lignocellulosic biomass [13-15]. Although this method could dramatically increase enzymatic hydrolysis rates and yields, much investment for corrosion-resistant and high-pressure reactors is needed, and degradation products that would impair downstream fermentation were also formed during this process. Hence, pretreatment still remains one of the most costly steps in cellulosic H_2_ production and is a significant barrier to commercialization of biohydrogen production from lignocellulosic biomass. From both economic and environmental perspectives, biological pretreatment using a lignin-degrading fungus is attracting more and more attention as an alternative method to break the lignin barrier and disrupt the crystalline structure [16,17]. Compared to thermochemical pretreatments, fungal pretreatment could increase the enzyme accessibility and improve the digestibility of holocellulose at a lower cost and with a simpler-to-operate process, with no or reduced toxic compounds produced for subsequent fermentation [18].

Therefore, this study was undertaken to determine if fungal pretreatment could enhance H_2_ production by CBP performance of *Thermoanaerobacterium thermosaccharolyticum* M18. To confirm fungal pretreatment efficiency, two commonly used thermochemical pretreatments were also selected for comparison. The biodelignification characteristics of pretreated cornstalk were described at first. After that the pretreated cornstalks were fermented b*y T. thermosaccharolyticum* M18 directly, and the cellulase activity secreted by strain M18 and H_2_ production efficiency from the cornstalk under different pretreatments were evaluated. This study is the first to utilize fungal pretreatment for enhanced H_2_ production by a CBP process.

## Results

### Biochemical and structural features of pretreated cornstalk

Fungal pretreatment using *Phanerochaete chrysosporium* can reduce the lignin content and crystallinity of lignocellulosic biomass effectively to aid more efficient enzymatic saccharification with either commercial or crude cellulase [18-20]. However, the application of fungal pretreatment for enhanced H_2_ production by consolidated bioprocessing has not been studied.

In this research, the objective of the pretreatment is to break the lignin seal and disrupt its crystalline structure to make cellulose more accessible for cellulase. More specifically, an appropriate pretreatment is desired so that lignin is degraded to the maximum while cellulose and hemicellulose are still retained [21-23]. In order to evaluate the feasibility of *P. chrysosporium* pretreatment for cornstalk prior to fermentation, the chemical compositions of cornstalk after fungal pretreatment were compared with other two typically thermochemical pretreatments (alkaline pretreatment and diluted acid pretreatment). As shown in Table 1, 35.3% of the lignin content was removed in fungal pretreated cornstalk compared to the control (non-pretreated sample), whereas only 9.5% holocellulose was decreased. In other words, nearly 90% cellulose and hemicellulose in fungal pretreated cornstalk were retained and available for subsequent fermentation. Compared to fungal pretreatment, a similar amount of lignin removal was obtained (32.3%) when using diluted acid pretreatment, accompanied by largely holocellulose reduction of 55.9% (10.5% cellulose and 45.4% hemicellulose). A substantial amount of holocellulose (5.9% cellulose and 56.2% hemicellulose) reduction was also observed for alkaline pretreatment, although the loss of lignin reached the maximum among the tested pretreatments.

**Table 1:** **Compositions of cornstalk under different pretreatment methods**

**Pretreatment**	**Composition (%)** ^**a**^			**Solid yield (%)** ^**b**^	**Removal yield (%)** ^**c**^		
	**Cellulose**	**Hemicellulose**	**Lignin**		**Cellulose**	**Hemicellulose**	**Lignin**
Untreated	42.6 ± 1.2	28.8 ± 0.3	20.7 ± 0.1	-	-	-	-
Alkaline pretreatment	68.3 ± 1.5	21.4 ± 0.7	9.4 ± 0.2	58.7 ± 1.7	5.9 ± 2.0	56.2 ± 1.8	73.1 ± 3.3
Acid pretreatment	55.2 ± 0.9	22.8 ± 0.7	20.3 ± 0.3	69.2 ± 2.6	10.5 ± 0.8	45.4 ± 1.1	32.3 ± 1.0
Fungal pretreatment	47.7 ± 0.8	31.3 ± 1.5	15.5 ± 0.1	86.2 ± 1.1	3.3 ± 0.09	6.2 ± 0.3	35.3 ± 2.1

^a^Composition is shown as percentage of the solid fraction before and after pretreatment.

^b^Solid yield is shown as percentage of the initial amount of dry matter.

^c^Removal yield is shown as percentage of the amount in the initial material.

**Figure 1:**
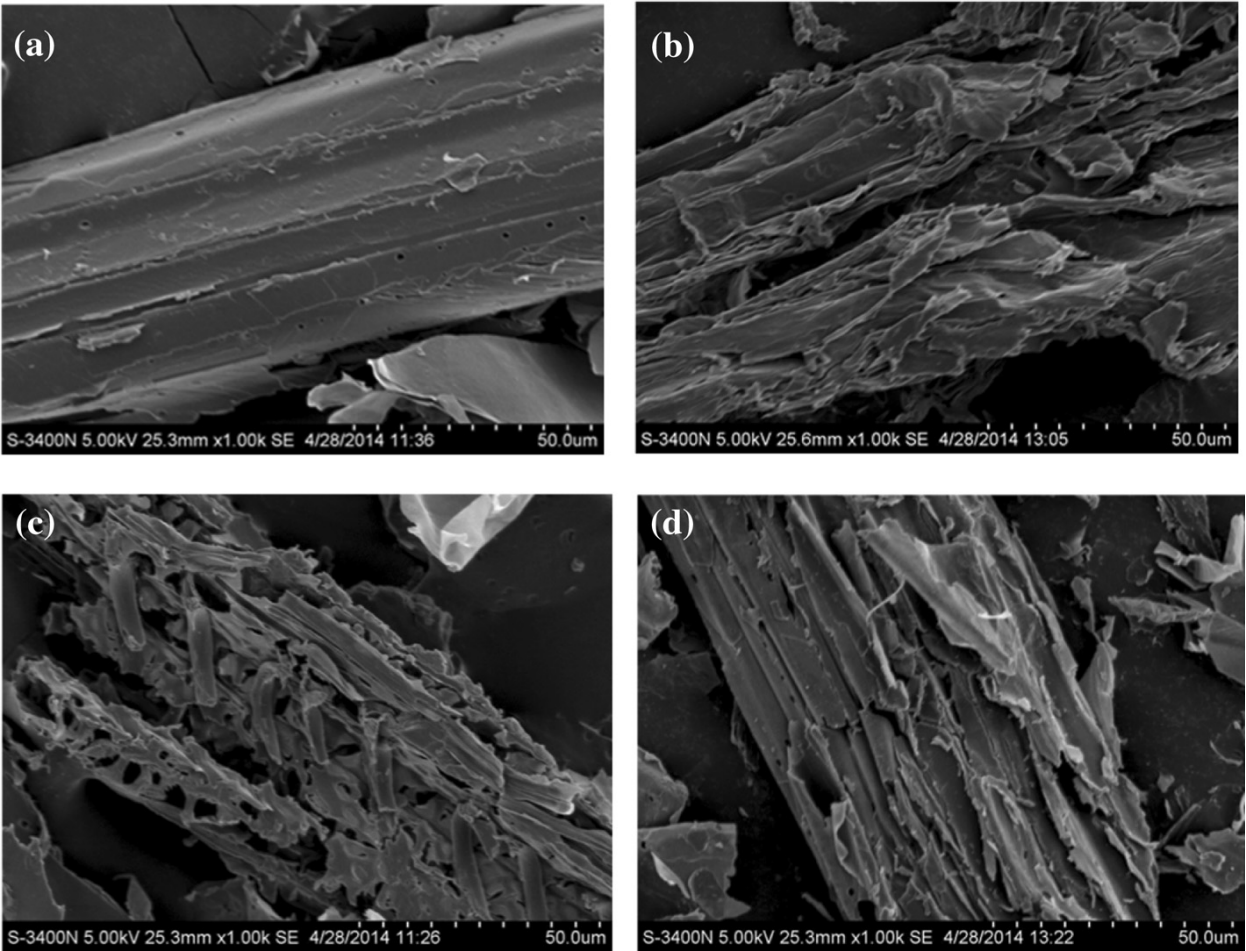
**Scanning electron micrographs (SEMs) of cornstalk under different pretreatment methods. (a)** Untreated, **(b)** alkaline pretreatment, **(c)** diluted acid pretreatment, **(d)** fungal pretreatment.

The morphological changes induced by different pretreatments were examined by scanning electron microscope (SEM) to provide insight into the structural modifications of cornstalk. Figure 1a shows the compact and rigid structure of unpretreated cornstalk. After alkaline pretreatment, the structure became loose, and the surface of the substrate seemed to be very smooth with the appearance of fragments (Figure 1b). Compared to alkaline pretreatment, significant destruction with a loose matrix was found on the cornstalk cell wall under diluted acid pretreatment; lots of erosion troughs and plenty of holes and cracks were exhibited in the surface of the pretreated cornstalk (Figure 1c). However, the SEM of the cornstalk pretreated by fungus showed a different morphology from that for alkaline and acid pretreated cornstalk. The surface damage of cornstalk by this pretreatment was minimized, the initial connected structure was partially loosed, and fewer cracks and scars were observed, as shown in Figure 1d. This appearance confirmed the partial degradation of lignin and better preservation of cellulose and hemicellulose compared with other two pretreatments, and these observations further demonstrated the chemical component analysis of pretreated cornstalk.

**Figure 2:**
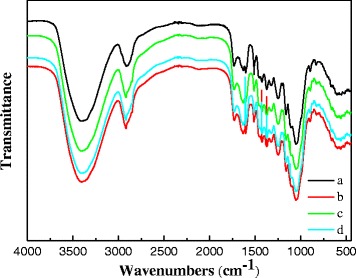
**FTIR spectra of cornstalk samples under different pretreatments.** (a) Untreated cornstalk, cornstalk pretreated with (b) alkaline pretreatment, (c) acid pretreatment, (d) fungal pretreatment.

Fourier transform infrared spectroscopy (FTIR) was applied as an analytical tool to further determine the chemical changes of cornstalk upon different pretreatment processes. As presented in Figure 2, the functional groups changes of pretreated cornstalks were particularly reflected in the fingerprint region of the absorbance spectra. The band at approximately 1512 cm^-1^ corresponded to aromatic skeletal vibration C = C of lignin [24]. The intensity of the peak at this wavelength was lower after all the three pretreatments than for untreated cornstalk, indicating that the content of lignin in cornstalk decreased after these pretreatments. Such results were in good accordance with chemical components analysis. The previous observations on spectra showed that the aromatic skeletal/C-O stretching ratio (R = I (1121 cm^-1^)/I (1078 cm^-1^)) in cellulose and hemicellulose could be quantified to represent the crystallinity of cellulose; the crystallinity decreases as the ratio increases [25]. The ratio increased from 1.09 (untreated cornstalk) to 1.68 (alkaline pretreatment), 1.33 (diluted acid pretreatment), and 1.53 (fungal pretreatment), respectively, showing that all the pretreatments adopted in this research could decrease the crystallinity of cornstalk efficiently. Also, it could be observed that the effect of fungal pretreatment by *P. chrysosporium* on cellulose crystallinity was even close to the alkaline pretreatment which is distinguished for crystallinity removal. These results indicate that the biodegradation capacity of fungal pretreatment for both lignin and crystalline zones of cellulose was comparable or even higher than that of the thermochemical pretreatments used in this research.

### Consolidated bioprocessing of pretreated cornstalk for hydrogen production

**Figure 3:**
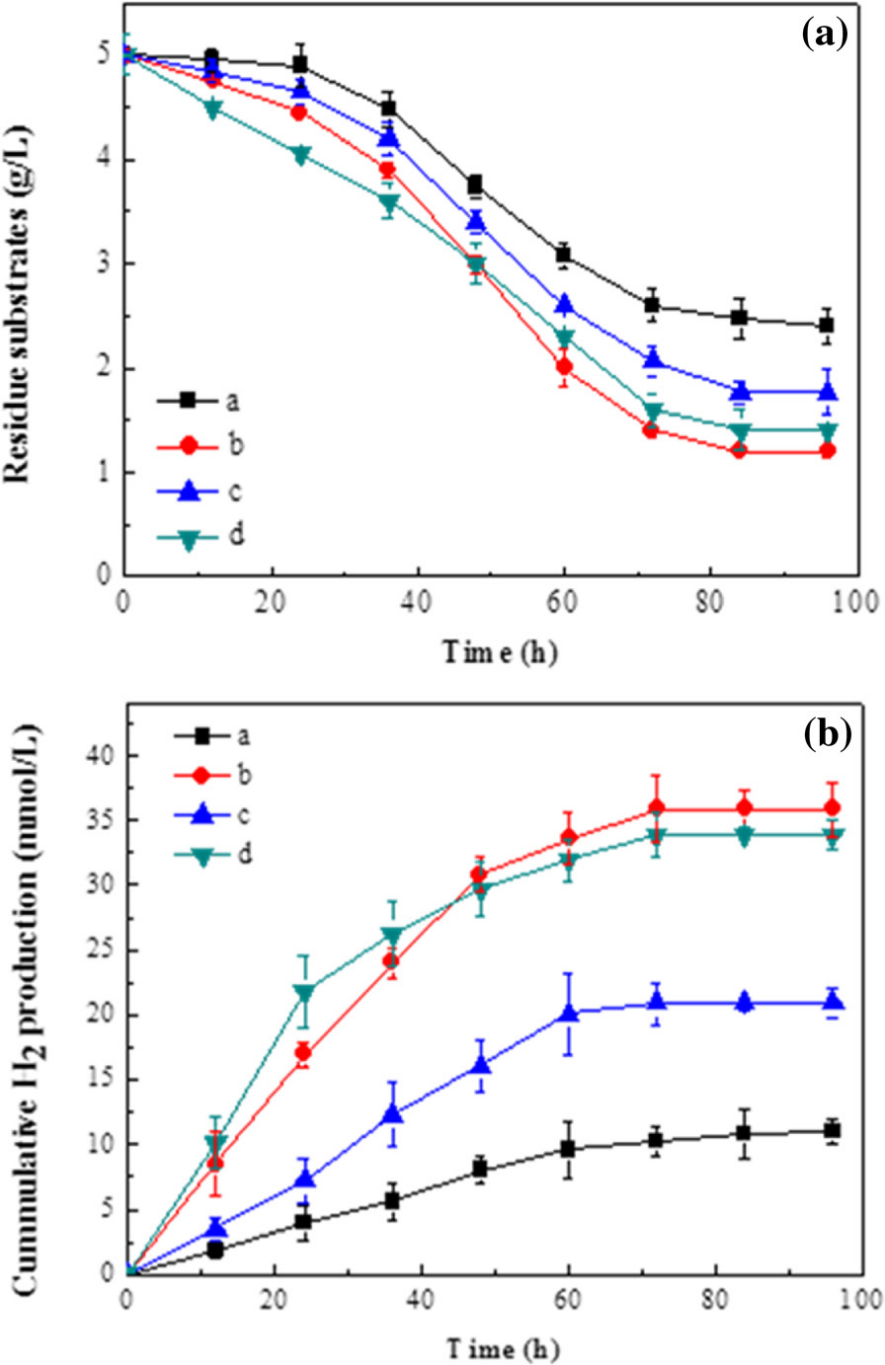
**Profiles of CBP for H**_**2**_ **production from cornstalk under different pretreatment methods. (a)** Residual weight of cornstalk, **(b)** amount of H_2_ produced. (a) untreated; (b) alkaline pretreatment, (c) diluted acid pretreatment, (d) fungal pretreatment.

**Figure 4:**
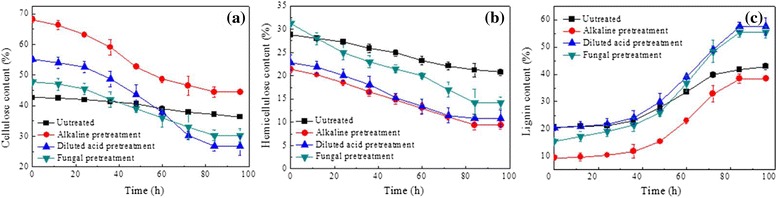
**Profiles of chemical components under different pretreatment methods. (a)** Cellulose content, **(b)** hemicellulose content, **(c)** lignin content.

Biological H_2_ potential tests from different pretreated cornstalk samples were performed using the candidate CBP strain *T. thermosaccharolyticum* M18 isolated by Cao *et al.*, [10]. As shown in Figure 3a, strain M18 displayed the most effective utilization on alkaline pretreated cornstalk, which resulted in more than 75% degradation of cornstalk after 96 h fermentation. Fungal pretreated cornstalk also supported appreciable growth of M18; nearly 72% of the substrate was degraded at the end of fermentation. Compared to using alkaline and fungal pretreated cornstalk as substrates, less diluted acid pretreated cornstalk was degraded. Although the disruption of the lignin-carbohydrate matrix enhances the digestibility of pretreated cornstalk, the degradation rates of cellulose and hemicellulose in resulting different pretreated cornstalk samples varied with different pretreatment conditions. As shown in Figure 4, the cellulose and hemicellulose components were decreased gradually accompanied by an increase of lignin for all substrates. However, the highest hemicellulose consumption rate was found in fungal pretreated cornstalk. This may attribute to the higher content of hemicellulose preserved in fungal pretreated cornstalk than the other pretreatments, since strain *T. thermosaccharolyticum* M18 has been reported to preferentially utilize hemicellulose [10].

Consistent with the degradation of substrate, the maximum cumulative H_2_ production of 35.9 mmol/L was obtained with the alkaline pretreatment (Figure 3b). It should be noted that fungal pretreatment could also largely enhance H_2_ production; a cumulative H_2_ production of 33.9 mmol/L was observed after 72 h fermentation, which was 2.9 times higher than that of the untreated cornstalk (10.9 mmol/L). Moreover, the results of analysis of variance (ANOVA) suggested that the cumulative H_2_ production results between alkaline pretreated and fungal pretreated cornstalk were statistically not significantly different (*P* >0*.*05*)*, which suggested that the application of fungal pretreated cornstalk was as efficient as alkaline pretreated cornstalk. In comparison, the cumulative H_2_ production of diluted acid pretreated cornstalk was decreased to 20.8 mmol/L, which could be attributed to the low hydrolysis of the substrate.

In order to understand and compare the utilization characteristics of different pretreated cornstalk samples by *T. thermosaccharolyticum* M18 for H_2_ production. The cumulative H_2_ production data depicted in Figure 3b were fitted with the Gompertz equation and the determined constants were listed in Table 2. The determination coefficient (R^2^) of over 0.98 for all the regressions confirmed that H_2_ production by strain *T. thermosaccharolyticum* M18 was well applied to the modified Gompertz model. As seen in Table 2, the order of enhancement for H_2_ production on the basis of the maximum potential H_2_ production was alkaline pretreatment > fungal pretreatment > diluted acid pretreatment > untreated, which was similar to the experimental data. Maximum H_2_ production rates (Rm) were also greatly improved after pretreatments. The highest Rm of 0.93 mmol L^-1^ h^-1^ was achieved by fungal pretreatment, which was nearly five times higher than that of the untreated (0.19 mmol L^-1^ h^-1^), while the effect of alkaline and dilute acid pretreatments on Rm was less obvious; the values were 0.79 mmol L^-1^ h^-1^ and 0.44 mmol L^-1^ h^-1^, respectively. Therefore, it could be assumed that hydrolysis of fungal pretreated cornstalk by *T. thermosaccharolyticum* M18 was much faster than hydrolysis of alkaline and diluted acid pretreated cornstalk. Moreover, the lag time of fermentation was shortened from 6.3 h to the lowest time of 4.4 h after fungal pretreatment, further implying that strain M18 preferred fungal pretreated cornstalk for H_2_ production. Based on a total H_2_ production and an uptake of cornstalk from different pretreatments, the yields obtained on using alkaline pretreatment and fungal pretreatment were in general roughly equivalent. In comparison, a lower yield of H_2_ was observed on dilute acid pretreated cornstalk (Table 3). In all cases, acetate and butyrate accounted for more than 80% of the total volatile fatty acid, accompanied by small amounts of ethanol, butanol, and propionate.

**Table 2:** **Kinetic parameters of cumulative H**_**2**_ **production for different pretreatments calculated from modified Gompertz equation**

**Pretreatment method**	**P (mmol H**_**2**_ **L** ^**−1**^ **culture)**	**R**_**m**_ **(mmol L** ^**−1**^ **h** ^**−1**^ **)**	***λ***	**R** ^**2**^
Untreated	11.5 ± 0.3	0.19 ± 0.01	6.3 ± 1.6	0.995 ± 0.001
Alkaline pretreatment	36.8 ± 0.6	0.79 ± 0.05	4.9 ± 1.4	0.992 ± 0.005
Acid pretreatment	22.0 ± 0.6	0.44 ± 0.03	5.3 ± 1.9	0.994 ± 0.008
Fungal pretreatment	33.6 ± 0.6	0.93 ± 0.09	4.4 ± 1.8	0.987 ± 0.003

**Table 3:** **Summary of fermentation parameters on different pretreated cornstalk samples using** ***T. thermosaccharolyticum*** **M18**

**Pretreatments**	**H**_**2**_ **yield** **(mmol H**_**2**_ **g** ^**−1**^ **substrate)**	**Concentration of end metabolites (mmol L** ^**−1**^ **)**				
		**Acetate**	**Butyrate**	**Ethanol**	**Butanol**	**Propionate**
Untreated	2.2 ± 0.03	9.1 ± 0.3	5.5 ± 0.1	2.1 ± 0.05	0.8 ± 0.03	0.5 ± 0.02
Alkaline pretreatment	7.2 ± 0.2	24.8 ± 0.4	13.3 ± 0.05	6.6 ± 0.03	1.0 ± 0.03	0.7 ± 0.03
Acid pretreatment	4.2 ± 0.1	17.2 ± 0.2	10.7 ± 0.1	4.8 ± 0.03	0.9 ± 0.04	0.6 ± 0.01
Fungal pretreatment	6.8 ± 0.2	23.2 ± 0.6	12.9 ± 0.2	5.7 ± 0.2	1.2 ± 0.01	0.6 ± 0.02

### Enzymatic activities of *T. thermosaccharolyticum* M18 under different pretreatments

**Figure 5:**
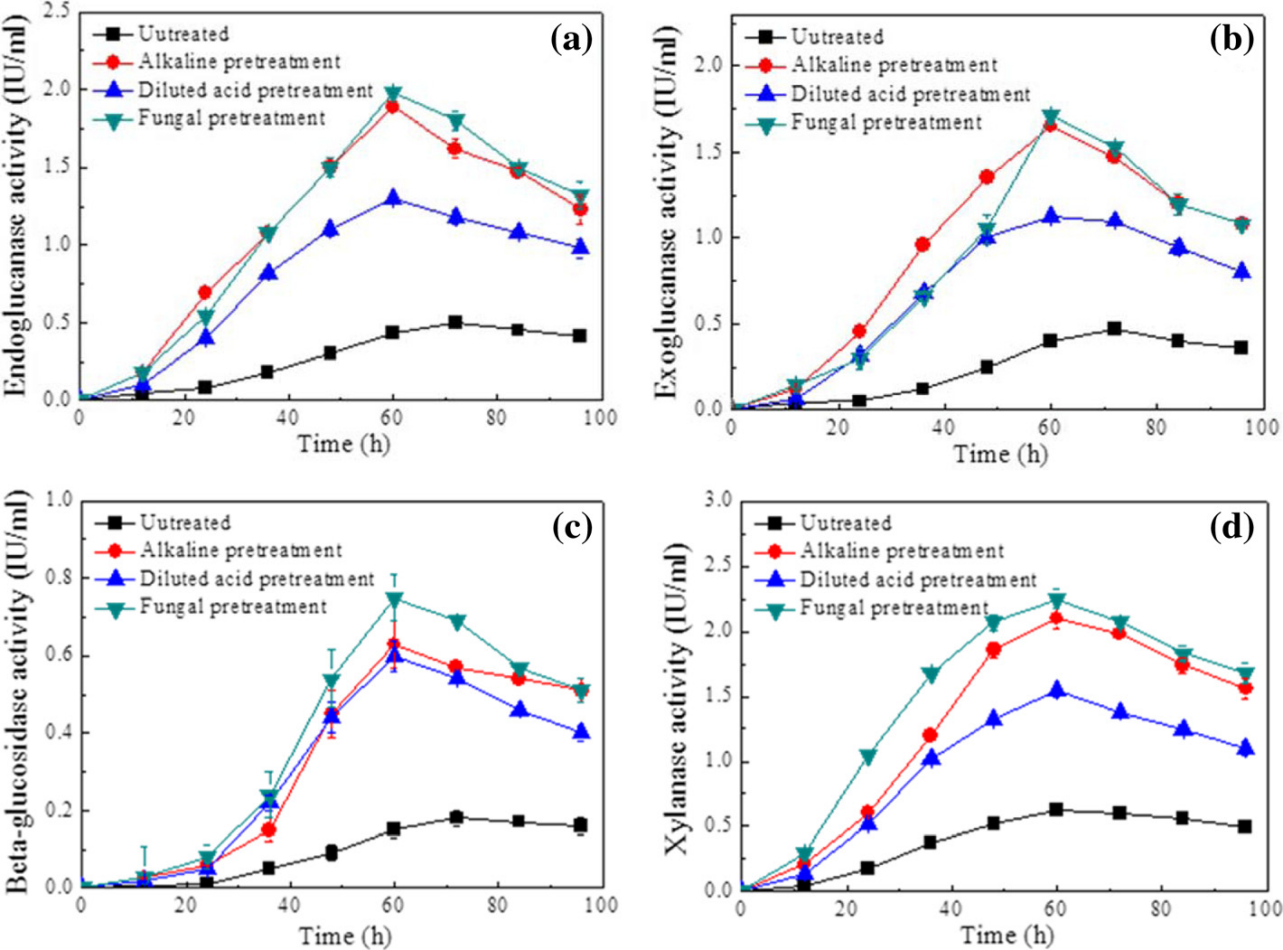
**Profiles of cellulolytic enzyme activity under different pretreatment methods. (a)** Endoglucanase activity, **(b)** exoglucanase activity, **(c)** beta-glucosidase activity, **(d)** xylanase activity.

The activities of enzymes during the whole fermentation process are shown in Figure 5, in accordance with the weight loss of substrate (as shown in Figure 4). Xylanase activity increased rapidly during the initial 48 h and reached a maximum level of 0.62 IU/mL, 2.1 IU/mL, 1.5 IU/mL, and 2.35 IU/mL, after 60 h fermentation when using untreated, alkaline, diluted acid, and fungal pretreated cornstalk as substrates respectively. However, the activity of endoglucanase, exoglucanase, and *β*-glucosidase were increased slightly less during the whole fermentation process; the order of maximum endoglucanase, exoglucanase, *β*-glucosidase activities were fungal pretreatment > alkaline pretreatment > acid pretreatment > untreated. Apparently, when fungal pretreated cornstalk was used as the substrate, the enzyme activity reached the highest, which may be an important explanation for why high relative degradation rate of biomass and yield of H_2_ was obtained on fungal pretreated cornstalk.

## Discussion

Two typical thermochemical pretreatments were selected for comparison with fungal pretreatment. Alkaline and diluted acid pretreatments have been widely used for pretreatment of lignocellulosic biomass for bioenergy production. Pretreatment with alkali such as NaOH could cause swelling of biomass, decrease the cellulose crystallinity, disrupt the lignin structure, and break the linkage between lignin and other carbohydrate fractions in lignocellulosic biomass, thus increasing the susceptibility of cellulose to enzymes [26-30]. However, during the pretreatment, lots of alkali is consumed, and the resulting alkali solution cannot be recovered due to the dissolution of lignin. The dissolved substances are toxic to the fermenting organisms and also cause pollution to the environment. Dilute sulfuric acid, commonly used as the acid of choice, is usually mixed with lignocellulosic biomass to solubilize lignin and hemicellulose so as to increase the accessibility of the cellulose in the biomass [30-32]. Nevertheless, lignocelllulosic biomass subjected to dilute acid pretreatment is hard to ferment directly because of the presence of fermentation inhibitors. In addition, corrosion caused by diluted acid pretreatment with sulfuric acid mandates expensive construction material. Compared to thermochemical pretreatments, fungal pretreatment reduces the recalcitrance of lignocellulosic biomass by lignin-degrading microorganisms, and no inhibitors are generated during the pretreatment process; thus, fungal pretreatment potentially provides an environmentally friendly and energy-efficient pretreatment technology for biofuel production [33]. The only problem to be discussed is the pretreatment efficiency of fungal pretreatment.

In order to understand the feasibility of using fungal pretreatment to improve H_2_ production in consolidated bioprocessing from cornstalk, two typical pretreatments of alkaline and diluted acid were used as a comparison in terms of the biodegradation characteristics of pretreated substrate, potential of H_2_ production, and cellulolytic enzyme activities. The results showed that alkaline, diluted acid, and fungal pretreatments all had a major impact on these parameters. The difference in the amounts of H_2_ produced from different pretreatments might be due to the differences in the ensuing lignocellulose structures, the constitutions after pretreatment, and their effect on cellulase and xylanase activities. Among the alternative pretreatments, alkaline pretreatment resulted in the highest lignin removal and cellulose conversion. However, the loss of hemicellulose resulted in the reduction of holocellulose content, which in turn led to a great waste of biomass and thus affected the substrate utilization efficiency. Taking into account the loss of biomass after alkaline pretreatment, the H_2_ yield obtained according to the initial cornstalk was only 3.9 mmol/g raw substrate. Fungal pretreatment could be considered as a moderate pretreatment method because of its lowest dry matter loss and the highest level of hemicellulose retained. Even though the delignification of fungal pretreatment was not as efficient as that of alkaline pretreatment, a similar yield of H_2_ was produced on fungal and alkaline pretreated cornstalk, implying that the same amount of material was degraded, but that the initial degradation rate of hemicellulose in fungal pretreated cornstalk was faster. This initial higher rate of degradation can be attributed to the higher amounts of readily accessible hemicellulose and the corresponding higher activity of cellulosic enzymes. As expected, the low loss of degradable biomass for fungal pretreatment resulted in the highest H_2_ yield according to the initial material; the value was up to 5.86 mmol/g raw cornstalk. Compared with the fungal pretreatment, a lot of hemicellulose was lost with lower removal of lignin for the acid pretreated cornstalk. Correspondingly, a low yield of H_2_ and low enzyme activities were obtained. These results indicated that although the removal of lignin is a crucial factor in determining the effect of pretreatment, retaining more degradable holocellulose during the pretreatment process is equally important. Overall, from the aspects of lignin removal, cellulose and hemicellulose preserved, and H_2_ yield, fungal pretreatment would be advisable as the optimal pretreatment method for enhanced CBP performance of lignocellulose to generate H_2_. The results presented here demonstrate for the first time the combination of fungal pretreatment with CBP to ferment cornstalk to H_2_, providing a new way for biological conversion of lignocellulosic biomass to H_2_ that is environmentally friendly and energy efficient.

## Conclusions

The complex structure of cornstalk was pretreated with several leading pretreatment methods for enhanced H_2_ production by CBP performance of *T. thermosaccharolyticum* M18. Major differences in substrate composition and H_2_ yield using different pretreatment methods were demonstrated. Of the methods examined, fungal pretreatment was considered the most suitable in contrast to other pretreatments with respect to the higher recovery of hemicellulose and cellulose, more efficient delignification, and higher yield of H_2_. Overall, fungal pretreatment could enhance H_2_ production directly from cellulosic materials and could be a good candidate for lignocellulosic biomass bioconversion processes.

## Methods

### Raw material

The cornstalk used in this research was collected from the suburb of Harbin, Heilongjiang province, China. Prior to use, the cornstalk was dried in the air and then ground to pass through a sieve with a 40-mesh (0.425-mm) screen. After that it was dried at 60°C and stored in plastic bags at room temperature to avoid possible interference.

### Pretreatment

The pretreatment methods used in this research were alkaline, diluted acid, and fungal pretreatments. Alkaline and diluted acid pretreatments were carried out in 500-mL flasks with a NaOH concentration of 2% (w/v) and H_2_SO_4_ concentration of 1.7% (v/v) for 120 min at 100°C and 121°C, respectively, with a solid-liquid ratio of 1:10. All remaining pretreated samples were centrifuged and thoroughly washed with deionized water to neutral, this washing step favors the removal of phenolic compounds, which are known to inhibit or deactivate enzymes [34]. Fungal pretreatment was carried out with the white rot fungus *Phanerochaete chrysosporium* (CGMCC5.776); the seed culture for pretreatment was prepared at 29°C, at a rotation speed of 150 rpm for 3 d. Then fungal pretreatment was performed in solid state using a 250-mL flask containing 10 g of raw cornstalk and seed culture at a moisture content of 70% at room temperature for 15 days as described by Zhao *et al.* [18]. Solid fractions were dried at 40°C for 24 h and then kept at 4°C for further chemical analysis and CBP process.

### Biohydrogen potential tests

Treated and untreated samples were digested in batch anaerobic conditions. Strain *T. thermosaccharolyticum* M18 isolated from rotted wood crumb by Cao *et al*., [10] was used as H_2_ producer. Fermentations were conducted in 100-mL serum bottles with 50 mL culture medium containing (g/L): K_2_HPO_4_, 3.0 g; KH_2_PO_4_, 1.5 g; NaCl, 1.0 g; KCl, 0.2 g; MgCl_2_, 0.2 g; NH_4_Cl, 1.0 g; yeast extract, 2.0 g; peptone, 2.0 g; cysteine-HCl, 0.5 g; vitamin solution, 1.0 mL; trace element solution, 1.0 mL [35]; and raw or pretreated substrates, 5.0 g. The initial pH was set at 7.0. Then the culture was incubated at 60°C and 150 rpm in an orbital incubator shaker for 96 h. All biohydrogen potential tests were performed in triplicate.

### Analytical methods

The chemical composition of untreated and pretreated cornstalk before and after the fermentation was determined by the procedures outlined by the National Renewable Energy Laboratory (NREL) [36,37]. The microstructural changes in the cornstalk after different pretreatments were detected by scanning electron microscopic (SEM). The specimens which were mounted on stubs coated with gold using a sputter coater were observed under a SEM (JEOL JSM-840). To characterize the functional groups change in pretreated cornstalk, FTIR spectroscopy was carried out using a Magna-IR 750 (Nicolet Instrument Co., USA) as described by Zhao *et al*. [18]. Spectra were recorded between 4000 and 400 cm^-1^. The background spectrum of pure potassium bromide was subtracted from the sample spectrum.The activities of cellulases and xylanase secreted by M18 were analyzed by the method as described by Ghose [38]. All activities were expressed in International Units, one unit of activity corresponded to the quantity of enzyme hydrolyzing one mmol of substrate or releasing one mmol of reducing sugars per minute [39]. High performance liquid chromatography (HPLC) equipped with a refraction index detector (LC-10A, Shimadzu Corporation, Kyoto, Japan) was used to detect any glucose, xylose, arabinose, and cellobiose that may have existed in the supernatant of the culture broth [40].

The activities of cellulases and xylanase secreted by M18 were analyzed by the method as described by Ghose [38]. All activities were expressed in International Units, one unit of activity corresponded to the quantity of enzyme hydrolyzing one mmol of substrate or releasing one mmol of reducing sugars per minute [39]. High performance liquid chromatography (HPLC) equipped with a refraction index detector (LC-10A, Shimadzu Corporation, Kyoto, Japan) was used to detect glucose, xylose, arabinose, and cellobiose that may be existed in the supernatant of culture broth [40].

Gas products (H_2_ and CO_2_) were analyzed by gas chromatography equipped with a thermal conductivity detector, N_2_ was used as carrier gas, and the column was packed with Molecular Sieve 5 Å (102 G, Shanghai Analysis Instrument Company, China) [41]. The soluble metabolites were analyzed by gas chromatography (4890D, Agilent Corporation, Santa Clara, CA, USA) with a hydrogen flame-ionization detector (FID) as described before [42].

### Data analysis

In this research, cumulative H_2_ production curves with respect to time were obtained first from the H_2_ production experiments; then the modified Gompertz equation (Eq. (1)) [43] was applied to determine the H_2_ production potential, H_2_ production rate, and lag phase:

1 $$ H=\mathrm{P}\times \exp \left\{- \exp \left[\frac{{\mathrm{R}}_{\mathrm{m}}\cdotp \mathrm{e}}{\mathrm{P}}\bullet \left(\lambda -t\right)+1\right]\right\} $$

where *H* denotes the cumulative H_2_ production (mL H_2_ L^−1^ culture), *P* denotes the maximum potential H_2_ production (mmol H_2_ L^−1^ culture), ***λ*** denotes the duration of the lag phase (h), *Rm* denotes the maximum H_2_ production rate (mmol L^−1^ h^−1^), *t* denotes the incubation time (h), and e = 2.71828.

Analysis of variance (ANOVA) was performed on the date, and a Tukey’s post hoc test was conducted to compute significant differences among different pretreatment methods. A *P* value of less than 0.05 was considered statistically significant.

## Abbreviations

Avicel: Microcrystalline cellulose
CBP: Consolidated bioprocessing
FTIR: Fourier transform infrared spectroscopy
Rm: Maximum H_2_ production rate
SEM: Scanning electron microscopic
VFAs: Volatile fatty acids
